# Tryptophan Residues Are Critical for Portal Protein Assembly and Incorporation in Bacteriophage P22

**DOI:** 10.3390/v14071400

**Published:** 2022-06-27

**Authors:** Brianna M. Woodbury, Tina Motwani, Makayla N. Leroux, Lauren F. Barnes, Nicholas A. Lyktey, Sanchari Banerjee, Corynne L. Dedeo, Martin F. Jarrold, Carolyn M. Teschke

**Affiliations:** 1 Department of Molecular and Cell Biology, University of Connecticut, Storrs, CT 06269, USA; brianna.woodbury@uconn.edu (B.M.W.); motwani.tina@gmail.com (T.M.); makayla.leroux@uconn.edu (M.N.L.); sanchari.banerjee@uconn.edu (S.B.); corynne.dedeo@uconn.edu (C.L.D.); 2 Department of Chemistry, Indiana University, 800 East Kirkwood Avenue, Bloomington, IN 47405, USA; barneslf@iu.edu (L.F.B.); nalyktey@iu.edu (N.A.L.); mfj@indiana.edu (M.F.J.); 3 Department of Chemistry, University of Connecticut, Storrs, CT 06269, USA

**Keywords:** virus assembly, DNA packaging, dsDNA virus, terminase

## Abstract

The oligomerization and incorporation of the bacteriophage P22 portal protein complex into procapsids (PCs) depends upon an interaction with scaffolding protein, but the region of the portal protein that interacts with scaffolding protein has not been defined. In herpes simplex virus 1 (HSV-1), conserved tryptophan residues located in the wing domain are required for portal-scaffolding protein interactions. In this study, tryptophan residues (W) present at positions 41, 44, 207 and 211 within the wing domain of the bacteriophage P22 portal protein were mutated to both conserved and non-conserved amino acids. Substitutions at each of these positions were shown to impair portal function in vivo, resulting in a lethal phenotype by complementation. The alanine substitutions caused the most severe defects and were thus further characterized. An analysis of infected cell lysates for the W to A mutants revealed that all the portal protein variants except W211A, which has a temperature-sensitive incorporation defect, were successfully recruited into procapsids. By charge detection mass spectrometry, all W to A mutant portal proteins were shown to form stable dodecameric rings except the variant W41A, which dissociated readily to monomers. Together, these results suggest that for P22 conserved tryptophan, residues in the wing domain of the portal protein play key roles in portal protein oligomerization and incorporation into procapsids, ultimately affecting the functionality of the portal protein at specific stages of virus assembly.

## 1. Introduction

The assembly pathway of many double-stranded DNA (dsDNA) viruses begins with the formation of a protein shell known as a procapsid (PC) [1,2]. Procapsids are minimally composed of three essential proteins—coat, scaffolding and portal proteins [3]. Previous studies in bacteriophages P22 [4], T4 [5,6], SPP1 [7], λ [8] and Φ29 [9,10] as well as in herpes simplex virus 1 (HSV-1) [11] suggest that procapsid assembly is initiated by the portal protein, which is a 12-subunit complex that occupies a single vertex of the capsid [12]. In all tailed dsDNA bacteriophages studied to date, the portal protein is a multifunctional ring that forms a hollow conduit for DNA entry and exit and also serves as a docking site for the DNA packaging machinery and tail components.

**Figure 1:**
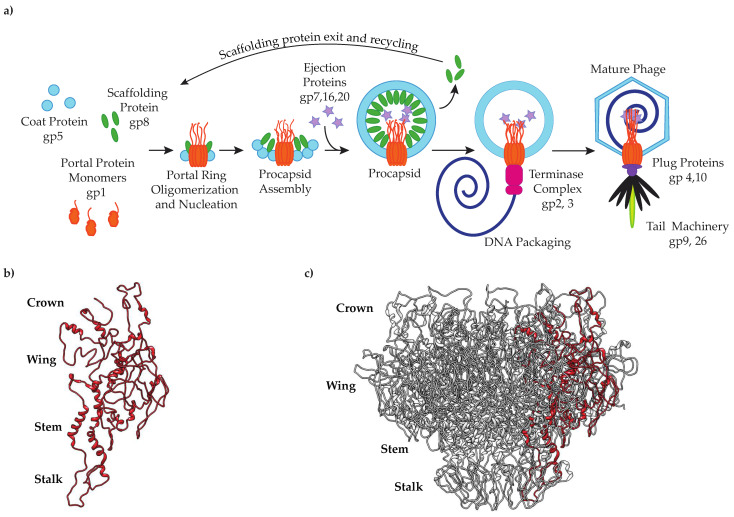
(**a**) Bacteriophage P22 assembly pathway; (**b**) single PC portal protein subunit; (**c**) PC portal protein complex (PDB: 5JJ1) [19].

Bacteriophage P22 is a well-characterized bacteriophage (phage) that has served as a model system for studying viral assembly since its discovery by Zinder and Lederberg in 1952 [13,14]. The assembly of bacteriophage P22 virions involves only 10 structural proteins (Figure 1a). To initiate assembly, scaffolding protein triggers the oligomerization of portal protein monomers into dodecameric rings [15]. Together, the dodecameric portal protein and 60–300 molecules of scaffolding protein form a nucleation complex upon which 415 molecules of coat protein polymerize to form an icosahedral shell [4,16]. The terminase complex, an ATP-dependent motor composed of large and small terminase proteins, docks onto the portal protein and powers the translocation of the genome into the procapsid through the portal channel [17]. DNA packaging is accompanied by the rearrangement of coat protein, the release of scaffolding protein and a conformational switch in the portal protein, resulting in the dissociation of the packaging motor. The tail factors then assemble onto the portal vertex to complete virus maturation [18,19]. Owing to the many years of study of phage P22, the genetic analyses, generation of mutants and their characterization are reasonably simple. Nevertheless, the results of mutants can be surprisingly pleiotropic because of the numerous protein:protein interactions involved at each step of assembly.

Despite their low sequence similarity, the portal proteins of dsDNA bacteriophages and herpesviruses are all structurally conserved, funnel-shaped oligomers with molecular masses that range from ~30 kDa to 80 kDa [12]. The highly conserved “portal-fold” consists of up to five domains: the stem, stalk, crown, wing and, for some bacteriophages including P22, the barrel [19,20,21,22,23,24,25,26,27] (Figure 1b,c). The structural organization of these regions lends a plasticity to the portal protein complex that facilitates long-range communication between its major domains and also governs the portal protein’s interactions with other viral proteins. For example, conformational changes propagated along the length of the portal complex regulate DNA packaging by altering the portal protein’s affinity for the terminase complex [3,22,28]. Structural adjustments of the portal protein have also been reported to stabilize the portal vertex within the capsid by promoting portal–capsid protein interactions [20,24,26,29,30,31,32]. In addition, conformational rearrangements of P22’s portal protein have been shown to influence in vitro portal–scaffolding binding [15].

Studies in HSV-1 revealed that highly conserved tryptophan residues within the portal protein (pU_L_6) facilitate portal–scaffolding protein interactions and that mutating these residues causes defects in portal protein incorporation and viral morphogenesis [33]. Interestingly, the conserved tryptophan residues of HSV-1 form a hydrophobic belt that encircles the portal protein wing domain [29], and this tryptophan belt is a feature shared by many tailed dsDNA bacteriophages such as P22, Φ29, SPP1, T4 and G20c [34,35]. Cryogenic electron microscopy (cryoEM) reconstructions of the P22 procapsid implicate the portal protein wing domain as a potential site for portal–scaffolding protein binding [36]. While there is structural and biochemical evidence that supports portal–scaffolding protein interactions in P22, the specific domains and residues in the portal protein that mediate binding to scaffolding protein have yet to be identified [15,36,37,38,39]. Given the structural similarities between the portal proteins of P22 and HSV-1, it is possible that the conserved tryptophan residues located within the wing domain of the P22 portal protein may also function in mediating protein–protein interactions [29,34].

In this study, we use site-directed mutagenesis to better understand the role of the tryptophan belt as it relates to portal–scaffolding protein binding in P22. We also present evidence that conserved tryptophan residues are critical for portal protein oligomerization in vitro and portal protein incorporation into procapsids in vivo.

## 2. Materials and Methods

### 2.1. Subcloning Portal Protein Gene into Twin-Strep-Tag Vector

Gene 1 (genbank accession DAA00985.1), which codes for the portal protein of bacteriophage P22 (genbank accession BK00583), was amplified by PCR and integrated into the pASG-IBA103 plasmid (IBA Lifesciences, Goettingen, Germany) as described in the StarGate^®^ Direct transfer cloning protocol (IBA Lifesciences) to generate the plasmid, pTM01. Cloning into this plasmid introduces a twin-strep tag at the C-terminus of the protein. The construct was verified by DNA sequencing and then transformed into *Salmonella enterica* serovar Typhimurium strain DB7136 (*leuA414^-^am hisC525^-^am sup^0^*) by electroporation [40]. Strain *Salmonella* DB7136 was used as the host for P22 growth.

### 2.2. Generation of His_6_-Tagged and Twin-Strep-Tagged Portal Protein Variants

Site-directed mutagenesis was used to introduce mutations into full-length gene 1 carried by two plasmids, pTM01 (this study) and pET21b (a kind gift of Dr. Peter Prevelige). The codons for residues 41, 44, 207 and 211 were changed from TGG to GCG to create amino acid substitutions W41A, W44A, W207A and W211A and generate the plasmids pTM01-W41A, pTM01-W44A, pTM01-W207A, pTM01-W211A as well as pET21b-1W41A, pET21b-1W44A, pET21b-1W207A and pET21b-1W211A. The codons for residues 41, 44, 207 and 211 were also changed from TGG to CAT, TTT or TAT to create the his, phe or tyr substitutions, respectively, in the pTM01 plasmid. All recombinant plasmids were confirmed by DNA sequencing and transformed into *Salmonella* DB7136 (pTM01 plasmids) or *Escherichia coli* strain BL21 (DE3) (pET21b plasmids) for expression.

### 2.3. Efficiency of Plating (EOP) Assay

Expression of the portal protein genes from the various pTM01 plasmids transformed in *Salmonella* DB7136 allowed for in vivo complementation of a phage with an amber mutation in gene 1 at codon 5 (1^-^*am*E5, referred to as 1^-^*am*), a generous gift from Peter Prevelige [39], or with WT P22. Cells carrying the various constructs were grown at 37 °C to mid-log phase, pelleted by centrifugation in a Sorvall SH-3000 rotor at 3829× *g* and resuspended in 1/10th the culture volume with LB media. Several drops of the cells were added to soft agar containing anhydrotetracycline (5 μg/L) and 1^-^*am* phage. The soft agar was poured onto LB agar containing ampicillin (100 μg/mL) and incubated overnight (O/N) at 30 °C or 37 °C. Plaques were counted to determine the phage titer of each portal protein variant relative to the titer of 1^-^*am* phages grown on *Salmonella* DB7136 cells expressing the wild-type (WT) portal protein genes from the pTM01 plasmid at 37 °C.

### 2.4. In Vivo Portal Protein Incorporation into PCs

*Salmonella* DB7136 cells transformed with pTM01 plasmids were grown at 30 °C or 37 °C to an optical density (O.D.) of ~0.4 in LB media containing ampicillin (100 μg/mL). The portal protein gene expression was induced with anhydrotetracycline (5 μg/L), and the cells were infected with 1^-^*am* phage at a multiplicity of infection (MOI) of 5. Cultures were grown an additional 4 h at 30 °C or 37 °C and then lysed with chloroform. Cell debris was removed by centrifugation at 14,200× *g* in a Sorvall F18-12 × 50 rotor for 10 min at 4 °C. The procapsids and phages were harvested at 38,725× *g* for 90 min at 4 °C. Cell pellets were re-suspended in 20 mM Tris-HCl (pH 7.6) with 100 mM MgCl_2_, shaken overnight at 4 °C and spun at 14,200× *g* for 10 min at 4 °C. The supernatants were transferred to sterile microcentrifuge tubes and stored at 4 °C.

Approximately 200 μL of each supernatant was layered onto linear 5–20% (*w*/*w*) sucrose gradients prepared with 20 mM Tris-HCl (pH 7.6), 100 mM MgCl_2_. Ultracentrifugation was executed at 105,000× *g* for 35 min in an RP55 S rotor. Fractions (100 μL) were collected from the top and analyzed by SDS-PAGE and Western blot.

### 2.5. Negative-Stain Transmission Electron Microscopy (TEM)

Aliquots (3 μL) of the peak procapsid-containing fractions and phage fractions from the sucrose gradients of the cell lysates (prepared above, Section 2.4) were adsorbed onto 300-mesh carbon-coated copper grids (Electron Microscopy Sciences, Hatfield, PA, USA) for 1 min at room temperature (~22 °C). The grids were washed with distilled water and stained with 1% uranyl acetate for 30 s at room temperature. The grids were blotted to remove excess stain and visualized in a FEI TecnaiG2 Spirit BioTWIN TEM at 68,000× magnification and 80 kV.

### 2.6. In Vivo Production of Procapsids

*Salmonella* DB7136 cells carrying the pTM01 plasmids were grown to an O.D. of ~0.4 in LB media containing ampicillin (100 mg/mL). The cells were simultaneously induced with anhydrotetracycline (5 μg/L) and infected with 1^-^*am*E5 13^-^*am*H101 phages (the 13^-^ *amber* mutation prevents cell lysis) at an MOI of 5. The infected cultures were grown for an additional 4 h then harvested by centrifugation at 7900× *g* in a Sorvall SLC-6000 rotor for 15 min at 4 °C. The cell pellets were resuspended in 20 mM sodium phosphate (pH 7.6) containing 20 mM MgCl_2_ and frozen at −20 °C. The pellet was processed as described previously, and the procapsids were loaded onto a 150-mL Sephacryl S1000 column (GE Healthcare, Chicago, IL, USA) equilibrated in 20 mM sodium phosphate (pH 7.6) at a flow rate of 0.20 mL/min at 4 °C [41]. The procapsid-containing fractions were pooled, pelleted by ultracentrifugation at 206,000× *g* for 40 min at 4 °C, and resuspended overnight (O/N) at 4 °C in 20 mM sodium phosphate (pH 7.6).

### 2.7. In Vitro Capsid Maturation and Stability Assays

In vivo generated and purified PCs were diluted to a final concentration of 1 mg/mL in 20 mM sodium phosphate buffer (pH 7.6). To test in vitro capsid expansion, PCs were incubated at temperatures between 22 °C and 72 °C for 15 min and then cooled on ice. The samples were analyzed on a 1% SeaKem LE agarose gel in 1× TAE buffer (40 mM Tris base, 20 mM acetate, 1 mM EDTA) for 70 min at 100 V. The gels were stained with Coomassie blue in 10% acetic acid.

In vitro capsid stability was assessed via urea titration. A 9 M urea stock solution was prepared in 20 mM sodium phosphate buffer (pH 7.6), and the refractive index was measured to confirm the concentration of the urea. Urea solutions with concentrations ranging from 0 M to 7 M were prepared in 20 mM sodium phosphate buffer (pH 7.6). The purified PCs were diluted to 0.5 mg/mL in each urea solution and incubated at room temperature (~22 °C) overnight. The samples were run on a 1% SeaKem LE agarose gel and stained, as mentioned above.

### 2.8. Fluorescent Western Blot

Sucrose gradient fractions containing procapsids were pooled and concentrated using a Millipore-Amicon ultra-0.5 centrifugal filter unit (molecular mass cutoff of 30 kDa). An equal amount of protein was loaded onto a 10% SDS-PAGE gel. Transfer to an Immobilon-PSQ membrane (Millipore, Burlington, VT, USA) was carried out using a TE22 transphor electrophoresis unit (Hoefer Scientific, Holliston, MA, USA) in transfer buffer (20 mM Tris base, 150 mM glycine (pH 8.3), 20% Methanol, and 0.01% SDS) overnight at 30 V and 0.1 A with stirring. The membrane was blocked with 10% nonfat milk in 1× Tris buffered saline (TBS) (20 mM Tris (pH 7.6) and 150 mM NaCl) for 1 h with gentle shaking. The blot was washed twice with 1× TBS/T (0.01% Tween 20 in 1× TBS) for 5 min. The StrepMAB-Classic antibody conjugated to Chromeo 488 (IBA Lifesciences) was pre-diluted 1:100 in enzyme dilution buffer (1× PBS, 0.2% BSA and 0.1% Tween 20) for storage and then diluted to a final working concentration of 1:1000 in antibody buffer (1% nonfat milk in 1× TBS/T). The membrane was incubated in the antibody solution for 2 h at room temperature with gentle agitation and no light and then washed three times in 1× TBS/T for 5 min. The blot was rinsed and stored in 1X TBS until visualization using a ChemiDoc MP imaging system (Bio-Rad, Hercules, CA, USA).

### 2.9. Portal Expression and Purification

The expression and purification of P22 portal protein monomers (PMs) and procapsid (PC) portal rings has been described previously [15,19,42]. To prepare PMs, the full-length His_6_-tagged portal protein gene was expressed in *E. coli* BL21 cells from the pET21b plasmid. The clarified cell lysate was applied to a 15-mL metal-affinity chromatography (IMAC) column (Talon Superflow, Clontech, San Jose, CA, USA). All chromatography was conducted at 4 °C. The eluate was dialyzed overnight against portal buffer (20 mM HEPES (pH 7.5), 70 mM NaCl, 3 mM β-mercaptoethanol and 1 mM EDTA) then loaded onto a 20-mL Q-Sepharose Fast Flow column (GE Healthcare) equilibrated in portal buffer. A linear 120-min gradient of portal buffer containing 1 M NaCl was used to elute the portal protein. Fractions of interest were pooled and concentrated using a Millipore-Amicon ultra-15 centrifugal filter device (molecular cutoff value of 30 kDa) at 3313× *g* and 4 °C in a Sorvall SH-3000 rotor. Concentrated PMs (<10 mg/mL) were clarified by ultracentrifugation at 23,900× *g* in a Sorvall S120-AT2 rotor for 15 min at 4 °C. To further resolve PMs and portal protein rings, the sample was injected onto a lab-packed Superose 6 Increase 16/70 column (GE Healthcare) at a flow rate of 0.20 mL/min. Fractions containing PMs were pooled, concentrated and ultra-centrifuged, as above. The supernatant was stored at −80 °C.

To assemble PC portal rings, the His_6_-tagged portal protein gene was expressed in *E. coli* BL21 cells and purified by IMAC using a 25-mL nickel column (Qiagen, Hilden, Germany). Fractions containing portal protein were dialyzed against the portal buffer. The purified protein was concentrated to ~100 mg/mL using a Millipore-Amicon centrifugal filter device (molecular mass cutoff of 30 kDa), as above, and incubated at room temperature (RT) for 48 h to facilitate oligomerization. Aggregates were removed by ultracentrifugation at 100,000× *g* for 35 min at 4 °C in a Sorvall S120-AT2 rotor. The supernatant was applied onto a Superose 6 Increase 16/70 column. Fractions were visualized on a NativePAGE 4–16% bis-Tris gel (InVitrogen, Waltham, MA, USA), and those containing portal protein rings were pooled for concentration, as above. The concentrated protein was ultra-centrifuged at 4 °C for 15 min at 23,900× *g*, and the supernatant was re-loaded onto the size exclusion column for further purification. PC portal rings were collected, concentrated and ultra-centrifuged prior to storage at −80 °C.

### 2.10. Circular Dichroism (CD) of PMs and PC Portal Rings

Purified PMs were prepared to 0.20 mg/mL in 20 mM phosphate buffer. CD spectra were acquired using a 0.1 cm path length quartz cuvette (Starna Cells, Inc., Atascadero, CA, USA) on a Pi-Star 180 spectropolarimeter (Applied Photophysics, Leatherhead, UK). Wavelength scans were completed between 195 nm and 320 nm at 20 °C with 1 nm intervals, a bandwidth of 2 nm and a time-per-point averaging of 15 s.

The CD spectra of PC portal rings were collected using a Chirascan V100 spectrometer (Applied Photophysics, Leatherhead, UK). PC portal rings were diluted with distilled water to 0.20 mg/mL in a 0.1 cm path length quartz, and the spectra were collected from 200–260 nm at 20 °C with 1 nm intervals, a bandwidth of 1 nm and a time-per-point averaging of 5 s. The thermal denaturation of PC portal rings was performed in a 0.1 cm path length quartz cuvette, and data were collected from 25–80 °C in 1 °C steps and with 1 nm intervals, a bandwidth of 1 nm and a time-per-point averaging of 1 s.

### 2.11. Proteolytic Digest

The proteolytic digestion of WT PMs and portal rings has been described previously [43]. WT and mutant PMs and PC portal rings were diluted to 0.11 mg/mL in portal buffer and digested with 0.005 U/mL of α-chymotrypsin (Sigma, St. Louis, MO, USA) at room temperature or 37 °C. Aliquots were taken at defined time points and then added to sample buffer containing 20 mM PMSF and immediately heated to 90 °C for 5 min. The proteolytic cleavage was assessed by SDS-PAGE (12.5%) and Western blot. The fluorescent Western blot was performed as described above using a 6X-His tag monoclonal antibody (HIS.H8) conjugated to DyLight 680 (ThermoFisher, Waltham, MA, USA).

### 2.12. Binding of Purified PC Portal Rings to L-Terminase and gp4

Full-length L-terminase (gp2) and gp4 genes were expressed in *E. coli* BL21 cells and purified by IMAC using a 25-mL nickel column as described previously [19]. To perform the pull-down assay, L-terminase and gp4 were diluted in coupling buffer (20 mM HEPES (pH 7.5) and 0.5 M NaCl) and individually incubated with CNBr-activated Sepharose 4B (Sigma) overnight at 4 °C using an end-over-end mixer. The coupled resin was blocked with 1 M ethanolamine and washed extensively according to the manufacturer’s protocol. For the binding of PC portal rings to immobilized L-terminase, 100 μg of WT or mutant PC portal rings was incubated with 25 μL of CNBr-coupled beads and 1 mM AMP-PNP (Roche, Mannheim, Germany) for 30 min at room temperature. The resin was washed with coupling buffer and spun down by centrifugation at 3380× *g* for 5 min. The supernatant was removed and the wash procedure repeated at least 3 times to remove unbound protein. After the final wash, the CNBr-coupled beads were resuspended in 25 μL of sample buffer, and 5 μL was loaded onto an SDS-PAGE gel for visualization. The same procedure was repeated for gp4, except AMP-PNP was omitted.

### 2.13. CDMS

Charge detection mass spectrometry is a single particle technique where the mass-to-charge ratio (*m/z*) and charge (*z*) are simultaneously measured for each ion [44,45,46]. The *m*/*z* and *z* are then multiplied to give the mass of the ion.

Purified PC portal rings were stored at −80 °C until ready for analysis. Samples were thawed and buffer exchanged using Micro Bio-Spin columns (Bio-Rad, 7326221) into 100 mM ammonium acetate (Honeywell, Charlotte, NC, USA, 631-31-8) solution. The exchanged samples were introduced into a homebuilt charge detection mass spectrometer via nano-electrospray ionization (Advion TriVersa NanoMate, Ithaca, NY, USA) [47,48,49,50]. The ions enter the CDMS instrument via a metal capillary; they then travel through three regions of differential pumping before entering a dual hemispherical deflection energy analyzer (HDA). In the HDA, ions with a narrow band of energies (around 100 eV/z) are selected and allowed to travel into an electrostatic linear ion trap (ELIT). A conducting cylinder is located in the center of the ELIT. The potentials on the endcaps of the ELIT can be switched between transmission and reflection modes, causing trapped ions to oscillate back and forth through the detection cylinder. When ions enter the cylinder, they induce a charge which is detected by a charge-sensitive amplifier. This signal is amplified, digitized and analyzed by fast Fourier transforms. The amplitude of the signal is proportional to the charge of the ion, while the oscillation frequency is related to its *m/z*. Thousands of ions are measured and binned into a histogram to give a mass spectrum.

## 3. Results

### 3.1. Residues in Portal Protein Tryptophan Belt Are Required to Generate Infectious Virions

**Figure 2:**
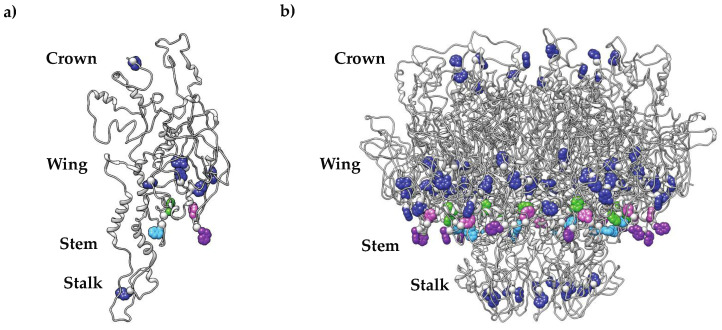
Tryptophan residues in the portal protein wing domain targeted for site-directed mutagenesis. Residues W41, W44, W207 and W211 present in the wing domain are colored green, light blue, purple and pink, respectively. All other tryptophan residues are colored dark blue. (**a**) Single PC portal protein subunit; (**b**) PC portal protein complex (PDB: 5JJ1).

In the tailed dsDNA phages, herpesviruses and adenoviruses, scaffolding protein is required for the incorporation of the portal protein into procapsids (PCs) and, ultimately, for the production of infectious virions [29,30,33]. In bacteriophage P22, scaffolding protein also induces the polymerization of portal protein [15]. In HSV-1, tryptophan residues in the portal protein (pU_L_6) wing domain are essential for scaffolding protein interactions [33]. The strong structural homology among portal proteins suggests that tryptophan residues located within the tryptophan belt of P22’s portal protein (product of gene 1; gp1) could be of similar significance. To determine if the tryptophan belt residues of P22’s portal protein are required to generate infectious phages, single amino acid substitutions were introduced at positions 41, 44, 207 and 211 to change the trp residues to ala, his, phe and tyr (Figure 2, Table 1).

WT and the mutant portal proteins were tested for their ability to complement a 1^-^amber (1^-^*am*) phage. The 1^-^*am* strains of P22 do not produce full-length portal protein from the phage genome and, as a result, do not yield infectious virions unless complemented by expression of a functional gene 1 from a plasmid. Plaques form only if the plasmid-encoded portal protein could support the growth of the 1^-^*am* phage. Only the 37 °C data are shown in Table 1 since the results at 30 °C were similar. The trp to ala, his, phe and tyr substitutions at each site led to decreases in relative titer from 5 to 10,000-fold (Table 1). The results suggest that tryptophan residues located in the tryptophan belt are essential for the generation of infectious phage particles, leading to lethal phenotypes regardless of whether the amino acid change was conservative or non-conservative.

### 3.2. Some Tryptophan Belt Residues Are Important for Portal Protein Incorporation into PCs In Vivo

To determine if the lethal phenotype of the tryptophan substitution mutants was due to failure to incorporate the portal protein into assembling PCs in vivo, *Salmonella* DB7136 cells transformed with pTM01 plasmids (described in the Materials and Methods) that express either full-length WT or variant portal genes were simultaneously induced with anhydrotetracycline (5 μg/L) and infected with gene 1 amber (1^-^*am*) phage at 30 °C or 37 °C. After 4 h, the cultures were lysed with chloroform and the clarified lysates were sedimented through 5–20% sucrose gradients. Fractions from the gradients were analyzed using several techniques.

The in vivo portal protein incorporation results for the W to H, F and Y portal protein mutants are summarized in Table 2. The W41F portal protein was not incorporated into PCs well at 37 °C; however, the titer for this mutant was only two orders of magnitude lower than WT. This result suggests that the W41F portal protein was functional when it was successfully incorporated. Since the effects of the alanine substitutions on phage titer and portal protein incorporation were either more severe or similar to other substitutions, the results for these variants are described further.

**Figure 3:**
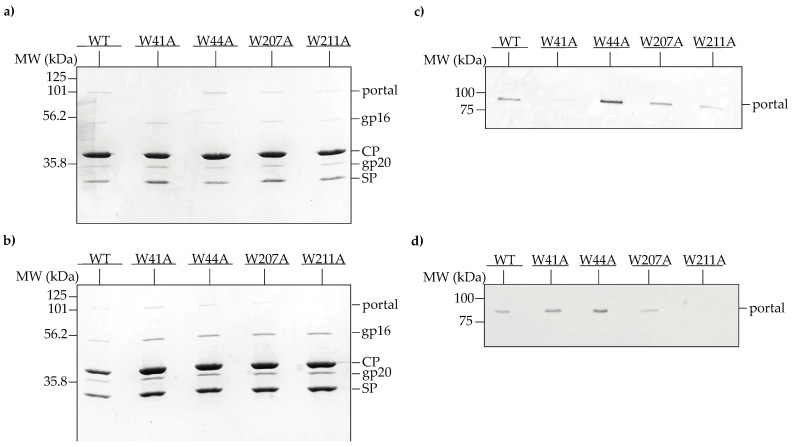
In vivo incorporation of WT and mutant portal protein into PCs at 30 °C (top row) and 37 °C (bottom row). (**a**,**b**) Cells carrying plasmids that express WT and variant portal genes upon induction with anhydrotetracycline were infected with gene 1 amber phage, then separated in 5–20% sucrose gradients and analyzed by 10% SDS-PAGE. The peak PC-containing fractions are shown above. Positions of portal protein, coat protein (CP), scaffolding protein (SP) and the ejection proteins, gp16 and gp20, are indicated on the right; (**c**,**d**) fluorescent Western blot of the corresponding sucrose gradient fractions using StrepMAB-Classic antibody.

The incorporation of the W to A portal protein mutants into PCs are shown in Figure 3. A band corresponding to portal protein was present in the PC region for all variants at 30 °C (Figure 3a). The W41A portal protein band was much less intense (very faintly visible to the eye), suggesting that it does not incorporate well compared to the other mutant portal proteins at 30 °C. At 37 °C, the WT, W41A, W44A and W207A portal proteins were each incorporated into PCs, suggesting that the substitutions did not disturb portal-scaffolding protein binding, though the band of W207A portal protein was less intense than the other samples (Figure 3b). However, no portal protein band was observed in the PC peak of the sucrose gradient of the W211A lysate at 37 °C (Figure 3b), indicating a defect in portal protein recruitment at this temperature. These results were further confirmed by Western blot analysis (Figure 3c,d).

The lethal phenotype of the W to A portal variants might be explained by the production of abnormal particles, since capsid assembly initiation starts with the portal protein [4]. Negative-stain electron microscopy of the peak PC-containing fractions from the 30 °C and 37 °C cell lysates showed normal-sized PCs for WT and each of the W to A portal protein variants (Figure S1a,b). Urea titrations of purified PCs were performed to determine whether the alanine substitutions affected capsid stability. The PCs assembled with WT and mutant portal proteins dissociated to monomers at 6 M urea, which suggests that the alanine substitutions did not destabilize the capsid (Figure S2a).

The bottom fraction (fraction 23) of each sucrose gradient, which contains some PCs, mature phages and any aberrant particles, was also visualized by negative-stain electron microscopy. Phages were not observed for the W41A variant at either temperature (i.e., 30 °C and 37 °C), and for the W211A variant, phages were not observed at 37 °C (Figure S1c,d). A mixture of phages and PCs were visualized for the W44A and W207A portal protein variants at both 30 °C and 37 °C (Figure S1c,d). Since the absence of mature virions could be explained by an inability to undergo capsid maturation, in vitro heat expansion assays were performed. These assays showed that PCs assembled with WT and mutant portal proteins underwent expansion at 67 °C (Figure S2b). Together, these results suggest that the variant portal proteins do not affect the coat protein shell. The lethal phenotype is therefore not caused by an assembly defect.

Finally, the dominance of the W to A substitutions on portal protein function was tested by plating WT P22 on *Salmonella* expressing WT or variant portal protein genes (Table 3). This should result in the formation of portal rings having both WT and variant subunits. If mixed portal rings lead to defects in phage production, the relative titer should decrease. Instead, no changes were observed, indicating that having some WT subunits in the portal ring is sufficient to rescue portal protein function.

### 3.3. In Vitro Assembled PC Portal Rings Are Primarily Dodecameric, except for the W41A Variant

**Figure 4:**
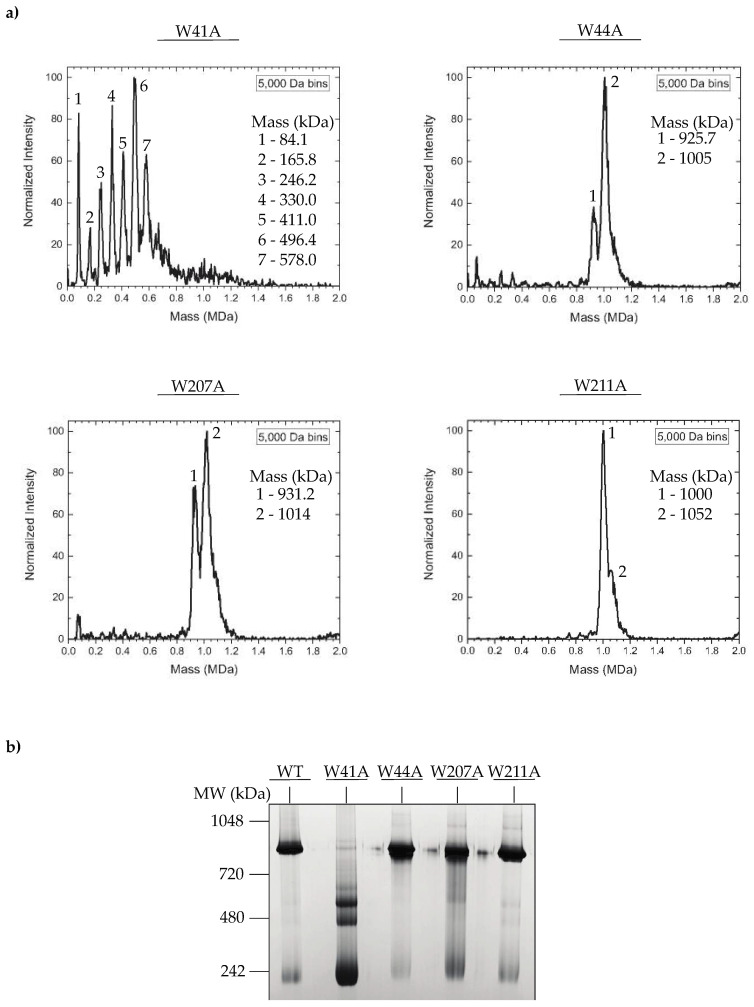
Oligomeric state of in vitro assembled PC portal rings. (**a**) Mass spectra of WT and variant PC portal rings measured by CDMS. The mass (in kDa) of each major peak is shown to the right. The expected mass of monomer portal protein is ~82.7 kDa for 11-mer is 910 kDa and for 12-mer is 993 kDa. (**b**) Native gel electrophoresis of WT or variant PC rings using native PAGE 4–16% bis-tris protein gel.

The decreased efficiency in portal protein incorporation for some of the W to A portal protein mutants could be caused by improper portal protein ring assembly. The portal proteins of dsDNA bacteriophages and herpesviruses are incorporated into PCs in vivo as dodecameric ring-like structures. The oligomeric states of ectopically expressed portal proteins can range from 11-mers to 14-mers [20,21,23,34,51]. However, in bacteriophage P22, charge detection mass spectrometry (CDMS), which measures the mass of native protein complexes [44,45,46] was previously used to demonstrate that in vitro assembled WT portal protein rings primarily exist as 12-mers [15]. To determine if there were mutant-specific defects in portal protein polymerization, we again used CDMS to probe the oligomeric state of in vitro assembled mutant PC portal rings. Briefly, the his_6_-tagged portal proteins were purified by metal affinity chromatography, then concentrated to ~100 mg/mL and incubated at room temperature (~22 °C) for 48 h, which generates PC portal rings [15,19]. The proteins were purified twice by size exclusion chromatography to eliminate aggregates and contaminating portal monomers (PMs). Previous studies have confirmed that the WT his_6_-tagged portal protein is functional in vivo and competent for ring assembly in vitro [43]. The mass spectrum of WT PC portal contained a 12-mer peak in addition to lower mass peaks, as previously observed (data not shown) [15]. While dodecameric rings were the dominant species in the spectrum of the W211A mutant, the spectra of the W44A and W207A portal proteins consisted of a mixture of 11-mer and 12-mer peaks (Figure 4a). In contrast, there were no peaks corresponding to 11-mers or 12-mers in the spectrum of the W41A mutant, but rather, a mixture of oligomers from monomers to 8-mers was observed (Figure 4a). These observations were further supported by a blue native bis-tris 4–16% gel in which all PC portal rings, except for the W41A mutant, migrated to the ~1-MDa position of WT PC portal rings (Figure 4b). The CDMS data indicate that alanine substitutions at positions 44, 207 and 211 of the portal protein did not prevent the assembly of dodecameric portal protein rings, whereas residue 41 was critical to the formation of stable, higher order dodecameric portal assemblies. In addition, while the W41A mutation was destabilizing, the W44A substitution promoted ring assembly, as it formed oligomers spontaneously at low protein concentrations (<10 mg/mL).

### 3.4. Alanine Substitutions Affect Portal Protein Folding into PC Portal Rings

Portal protein monomers (PMs) undergo structural rearrangements upon oligomerization into dodecameric rings, and these conformational changes can be observed as an increase in α-helical content [43]. Thus, to determine if the alanine substitutions interfered with protein folding, we evaluated the secondary structure of WT and variant PMs and PC portal rings by circular dichroism (CD). To prepare His_6_-tagged PMs, WT and mutant portal protein genes were expressed from a pET21b plasmid in *E. coli* BL21 cells and were purified using a combination of metal affinity, anion exchange and size exclusion chromatography. The PC portal rings were prepared as described above.

**Figure 5:**
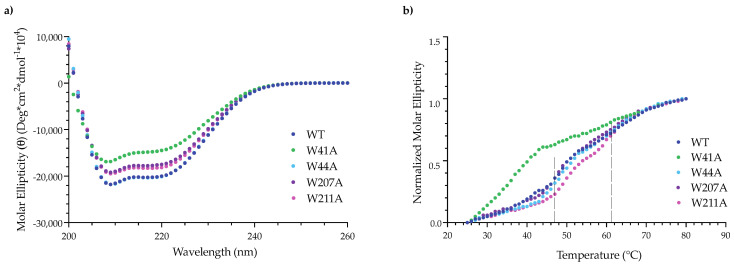
The effect of alanine substitutions in the wing domain of portal protein on the secondary and tertiary structure of PC portal rings. (**a**) CD spectra of in vitro assembled WT and mutant PC portal collected from 200 to 260 nm at 20 °C in dH_2_O; (**b**) thermostability of purified WT and mutant PC portal rings measured by CD at 222 nm from 25–80 °C in dH_2_O. The dotted lines indicate the midpoint of the two melting transitions previously observed in WT portal rings [43].

The CD spectra of the variant PMs were similar to the spectrum of WT PMs and were characteristic of a protein with a high α-helical content (Figure S3) [43]. For the mutant PC portal rings, there was a slight decrease in the secondary structure of the W44A, W207A and W211A PC portal rings when compared to the spectrum of WT PC portal rings (Figure 5a). The loss of α-helical secondary structure observed for the W41A PC portal rings was expected given the high monomer content of the sample [43].

Circular dichroism was also used to assess the thermodynamic stability of the in vitro assembled PC portal rings. Portal protein ring assembly is accompanied by the formation of a new cooperative folding unit that functions to stabilize the α-helical secondary structure of the protein. This new cooperative folding unit can be observed as two unfolding transitions in CD measured at 222 nm [43]. Thus, if the alanine substitutions have disrupted intra- or inter-subunit interactions, then variations in melting behavior may be seen. The W41A PC portal protein exhibited a single cooperative unfolding transition between 35 °C and 45 °C, as expected, since it is primarily monomeric [43]. The WT and other mutant PC portal rings displayed two melting transitions (Figure 5b). For WT, W44A and W207A, the first melting transition occurred between 46 °C and 53 °C and was followed by a second transition between 55 °C and 65 °C (Figure 5b). The melting curve for W211A was shifted to slightly higher temperatures, with the first unfolding transition from 48 °C to 53 °C and the second from 58 °C to 65 °C (Figure 5b), suggesting the W211A substitution enhanced the stability of the portal ring complex.

**Figure 6:**
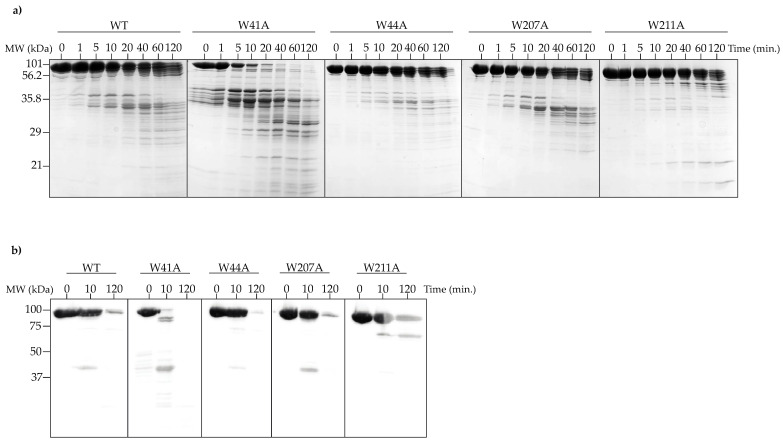
Tertiary structure of WT and mutant PC portal rings probed by proteolytic digestion with α-chymotrypsin at 37 °C. (**a**) 12.5% SDS-PAGE showing proteolytic digestion of PC portal rings. The 0 min lane shows the full-length portal proteins without protease added; (**b**) fluorescent Western blot using anti-6x-His Tag antibody showing proteolytic digestion of PC portal rings. The his-tag is on the C-terminus of the portal proteins.

To further test the effects of the tryptophan substitutions on the stability of PC portal rings, limited proteolysis experiments were performed. WT and mutant PC portal rings were incubated with α-chymotrypsin for 120 min at room temperature (~22 °C) or 37 °C. Samples were taken at various times and analyzed by SDS-PAGE. Only the data collected at 37 °C are shown, as the RT data were similar. An analysis of the SDS-PAGE gel indicates that after 20 min, the W41A PC portal protein was completely degraded into smaller peptides, which was an expected result given the high ratio of monomers to rings obtained after the purification of this mutant (Figure 6a). The WT and other variant PC portal rings were not completely digested even after 120 min (Figure 6a), and the respective patterns of cleavage for WT, W44A and W207A portal rings were similar, demonstrating that the same regions were available for digestion. In contrast, α-chymotrypsin did not efficiently degrade the W211A PC portal rings, as fewer peptide fragments were generated over the two-hour time course (Figure 6a). Western blot analysis revealed that the C-terminus of the W211A mutant was more resistant to proteolytic cleavage (Figure 6b). The results for WT PC portal rings were consistent with previously published data [43]. However, these data indicate that W211A PC portal rings were more stable than WT PC portal rings.

### 3.5. Portal Protein Variants Bind to L-Terminase and the Plug Protein, gp4

**Figure 7:**
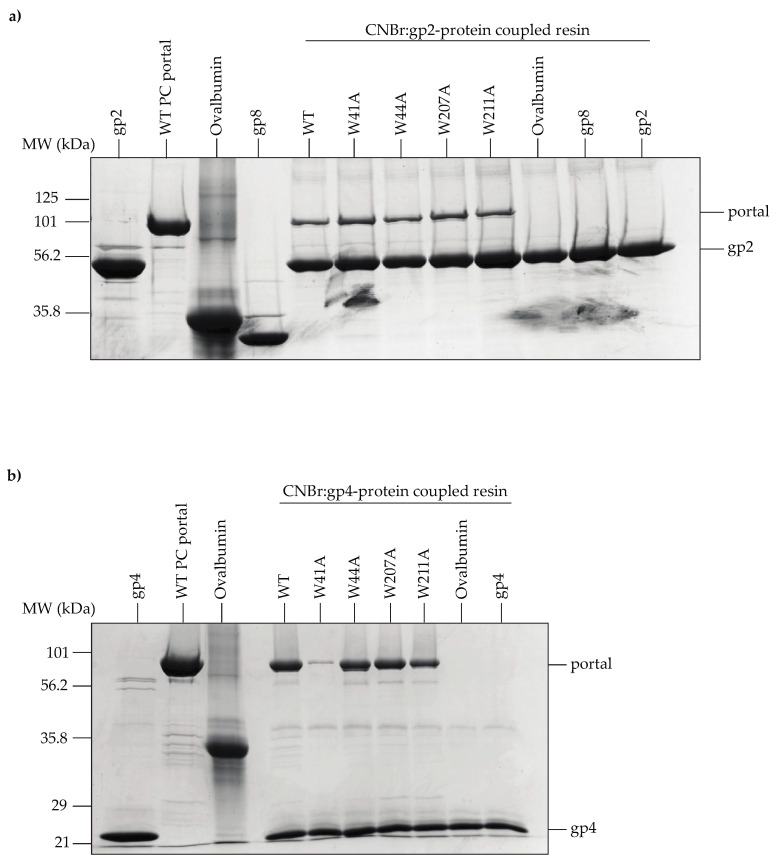
WT and mutant PC portal rings interact with large terminase (L-terminase; gp2) and gp4 immobilized on CNBr-activated Sepharose 4B. (**a**) SDS-PAGE analysis of PC portal rings binding to gp2-Sepharose; (**b**) SDS-PAGE analysis of PC portal rings binding to gp4-Sepharose. Ovalbumin and gp8 (P22 scaffolding protein) are negative controls.

The conformational rearrangements that occur within the clip region of the portal protein during phage maturation are known to affect its binding affinity for the L-terminase (gp2) subunit of the packaging motor [19]. Casjens et al. (1992) found that residues 64 and 303 located within the portal wing domain of P22’s portal protein regulate the amount of DNA that is packaged into PCs, as point mutations at these positions lead to overpackaging [3]. These data suggest that crosstalk between the wing and clip domains contributes to key functions of the portal such as DNA packaging. To determine if the tryptophan substitutions altered the portal protein’s interactions with L-terminase, we coupled purified gp2 to Sepharose 4B agarose beads and evaluated binding to WT and variant PC portal rings. All PC portal rings bound to the gp2-coupled beads (Figure 7a). The result for the W41A mutant was expected since it was previously established that gp2 can bind to PMs and PC portal rings in vitro [19].

Gp2 is displaced from the portal protein by the tail factor gp4 once genome packaging is complete [52]. Gp4 functions to stabilize the portal protein and protect against genome loss by acting as the head-to-tail adapter; hence, it is critical to the formation of infectious progeny virions [53]. We performed a pull-down assay using gp4-coupled agarose beads to assess whether the alanine substitutions affected the ability for gp4 to associate with PC portal rings in vitro. The W41A variant was the only mutant unable to bind gp4 (Figure 7b), which was an expected result based on previous work showing that gp4 does not interact with PMs in vitro [19,53].

The results from the pull-down assays suggest that the alanine substitutions have not induced conformational changes in the portal protein that impact its ability to form a complex with gp2 or gp4 in vitro.

## 4. Discussion

Portal proteins perform essential functions throughout the viral life cycle and often depend upon interactions with other viral proteins to accomplish those functions. For instance, it is widely accepted that the portal proteins of dsDNA bacteriophages and herpesviruses interact with scaffolding proteins [7,8,9,10,15,29,33,37,38,54,55,56]. Recent in vitro assembly experiments in P22 found that (i) scaffolding protein drives the oligomerization of portal protein monomers into dodecameric rings [15] and (ii) the assembled portal protein rings act as a nucleation site for procapsid assembly [4]. CryoEM reconstructions of the P22 procapsid provide further support for a direct interaction between portal and scaffolding proteins [31,36]. Here, we investigated whether tryptophan residues located within the portal protein wing domain are important for portal-scaffolding protein binding. Our results suggest that these tryptophan residues may contribute to several portal protein functions but, in general, not to the binding of scaffolding protein.

### 4.1. The Tryptophan Belt Residues Are Not Critical for Binding to Scaffolding Protein but Are Essential for Viral Maturation

The tryptophan residues in the portal protein wing domain tryptophan belt are not responsible for scaffolding protein interactions (Table 4). However, the tryptophan belt may be involved in mediating interactions between the portal and coat proteins. While our in vitro experiments suggest that the portal protein alanine variants do not affect capsid stability or capsid expansion, inappropriate interactions between the variant portal proteins and coat protein may nonetheless account for the inability of in vivo assembled PCs to properly package DNA and mature into infectious virions. The tryptophan belt residues targeted in this study are positioned within two loop regions (residues 41 to 59 and 191 to 217) of the wing domain. These loop regions have been identified as a potential coat protein binding domain in P22 [15,31]. There are several hypotheses regarding the importance of portal–coat protein interactions in dsDNA viruses: (i) coat protein, along with scaffolding protein, may help to recruit the portal protein complex into PCs [15,57]; (ii) structural rearrangements of the portal and coat proteins that occur during viral maturation may stabilize the portal complex within the capsid [20,21,23,29,30,32]; (iii) the portal protein may drive capsid expansion [58,59] and (iv) portal-coat protein interactions may trigger conformational changes in the portal protein that regulate DNA packaging and/or the binding of plug proteins [26,31]. Thus, differences in portal-coat protein interactions have the potential to disrupt various stages of the viral life cycle.

### 4.2. In Vitro Characterization of Variant Portal Protein Rings Reveals Oligomerization Defect

Portal proteins are dynamic assemblies that undergo structural transformations during viral morphogenesis. Raman spectroscopy of the P22 portal protein revealed that the assembly of portal protein rings from monomers induces substantial changes in the local side chain environments of cysteine, tyrosine and tryptophan residues, with the most dramatic changes occurring in the region that corresponds to the inter-subunit interface [60]. In previous work, all four of the P22 portal protein cysteine residues (C153, C173, C283 and C516) were mutated to serine and characterized both in vitro and in vivo to understand the conformational rearrangements. The hydrogen-bonding interactions of the cysteine residues were shown to be critical to portal protein stability and dodecameric ring formation, and the in vitro assembly-related defects manifest in vivo as a decrease in phage production [61,62]. Our results demonstrate that tryptophan residues within the hydrophobic belt are also important for portal ring formation and stability. Residues W41 and W44 are in close proximity to the subunit interface in portal protein rings, and the effects of the substitutions suggest that hydrophobic interactions stabilize the inter-subunit interface. Although the W211A mutant is not buried within the subunit interface, it impacts the portal ring assembly pathway since it forms exclusively 12-mer portal rings (Figure 4) that are more stable than WT portal rings (Figure 6). Thus, differences in portal protein ring stability and formation contribute to the lethal phenotype and suggest that a Goldilocks level of portal stability is required for proper function.

### 4.3. DNA Packaging Is Affected by Substitutions in the Tryptophan Belt

H/D exchange data of the P22 portal protein indicate that residues 41 and 44 are located within a dynamic region of the protein that coordinates DNA packaging via crosstalk with the stem helices [28]. No empty heads with tails were observed in our micrographs, indicating there is not a defect in association with the plug proteins, which would result in the DNA falling out of the matured heads. Though the in vitro assembled W to A rings were able to interact with gp2 and gp4, the purified PC particles from phage-infected lysates are filled with scaffolding protein and have portal protein, suggesting that DNA packaging is not initiated with high efficiency in vivo. While the variant PC portal rings may allow for the assembly of a portal-gp2 complex, subtle conformational changes within the portal protein in PCs likely blocks proper DNA packaging by impairing crosstalk between the portal protein stalk domain and gp2.

We conclude that the tryptophan residues interrogated in this study (i) can affect portal protein assembly into dodecameric rings and (ii) the portal rings assembled with subtle changes to stability are unable to coordinate with gp2 to initiate DNA packaging. The data presented here highlight the importance of communication between distant regions of portal protein that govern terminase protein interactions.

We dedicate this article to the memory of Lindsay Black, a pioneer in the study of bacteriophage portal protein structure and function [63,64].

## Figures and Tables

**Table 1:** Relative efficiency of plating of trp to ala, his, phe and tyr portal protein variants.

Portal Protein Variant	~Fold Decrease in Titer Relative to Complementation with WT Portal Protein at 37 °C ^a^
W41A	10,000
W41H	300
W41F	100
W41Y	100
W44A	10
W44H	15
W44F	15
W44Y	15
W207A	10
W207H	5 (35-fold decrease at 41 °C)
W207F	5 (50-fold decrease at 41 °C)
W207Y	200
W211A	100
W211H	70
W211F	30
W211Y	150

^a^ Reversion frequency of the gene 1 amber phage on the suppressor minus host DB7136 was 10^−7^. Depending on the temperature, complementation when the WT portal gene is expressed from plasmid pTM01 was ~60–90% as efficient as when growing the gene 1 amber phage on the amber suppressor host DB7155 (see Section 2).

**Table 2:** In vivo incorporation of trp to his, phe and tyr portal protein variants into procapsids.

Portal Protein Variant	Incorporation of Portal Protein into Procapsids In Vivo at 37 °C ^a^
W41H	Yes
W41F	No
W41Y	Yes
W44H	Yes
W44F	Yes
W44Y	Yes
W207H	Yes
W207F	Yes
W207Y	Yes
W211H	Yes
W211F	Yes
W211Y	Yes

^a^ Determined by analysis of Coomassie stained SDS gels of procapsids.

**Table 3:** Test of dominance of W to A portal protein variants on WT portal protein from phage.

Portal Protein Variant	Relative Fold Change in Titer for the Following Temperatures ^a^:	
	30 °C	37 °C
WT	1.0 ± 0.00	1.86 ± 0.29
W41A	1.31 ± 0.29	1.07 ± 0.45
W44A	2.06 ± 0.43	2.01 ± 0.31
W207A	0.93 ± 0.91	1.03 ± 0.83
W211A	0.98 ± 0.56	1.03 ± 0.40

^a^ The fold change in titer is relative to the titer when WT gene 1 was expressed from a plasmid and infected with WT P22 at 30 °C.

**Table 4:** Summary of results of the lethal W→A portal protein substitutions.

	WT	W41A	W44A	W207A	W211A
Portal protein incorporated into PCs in vivo at 30 °C, indicating ability to interact with scaffolding protein	Yes	Very little	Yes	Yes	Yes
Portal protein incorporated into PCs in vivo at 37 °C, indicating ability to interact with scaffolding protein	Yes	Yes	Yes	Yes	No
In vitro PC portal ring oligomeric state	12-mers + some monomers	Monomers + small oligomers	11- and 12-mers	11- and 12-mers	12-mers
Change in stability by circular dichroism temperature melts relative to WT PC portal rings	--	Destabilized, melt similar to WT PMs [43]	WT-like	WT-like	More stable
PC portal ring sensitivity to α-chymotrypsin	--	More digestion than WT	WT-like	WT-like	Less digestion

## Data Availability

Not applicable.

## Supplementary Materials

The following supporting information can be downloaded at: https://www.mdpi.com/article/10.3390/v14071400/s1, Figure S1: In vivo generated PCs and phages containing alanine-substituted portal proteins have normal morphologies; Figure S2: The alanine substitutions did not affect capsid stability or capsid expansion; Figure S3: Circular dichroism of WT and alanine-substituted portal protein monomers.
