# Supplementary material for: Tryptophan Residues Are Critical for Portal Protein Assembly and Incorporation in Bacteriophage P22
Source: Viruses. 2022 Jun 27;14(7):1400. doi: 10.3390/v14071400 (PMC9320234; doi:10.3390/v14071400)
Supplement: Supplementary file 1 [file viruses-14-01400-s001.zip › viruses-1711575-supplementary.pdf]

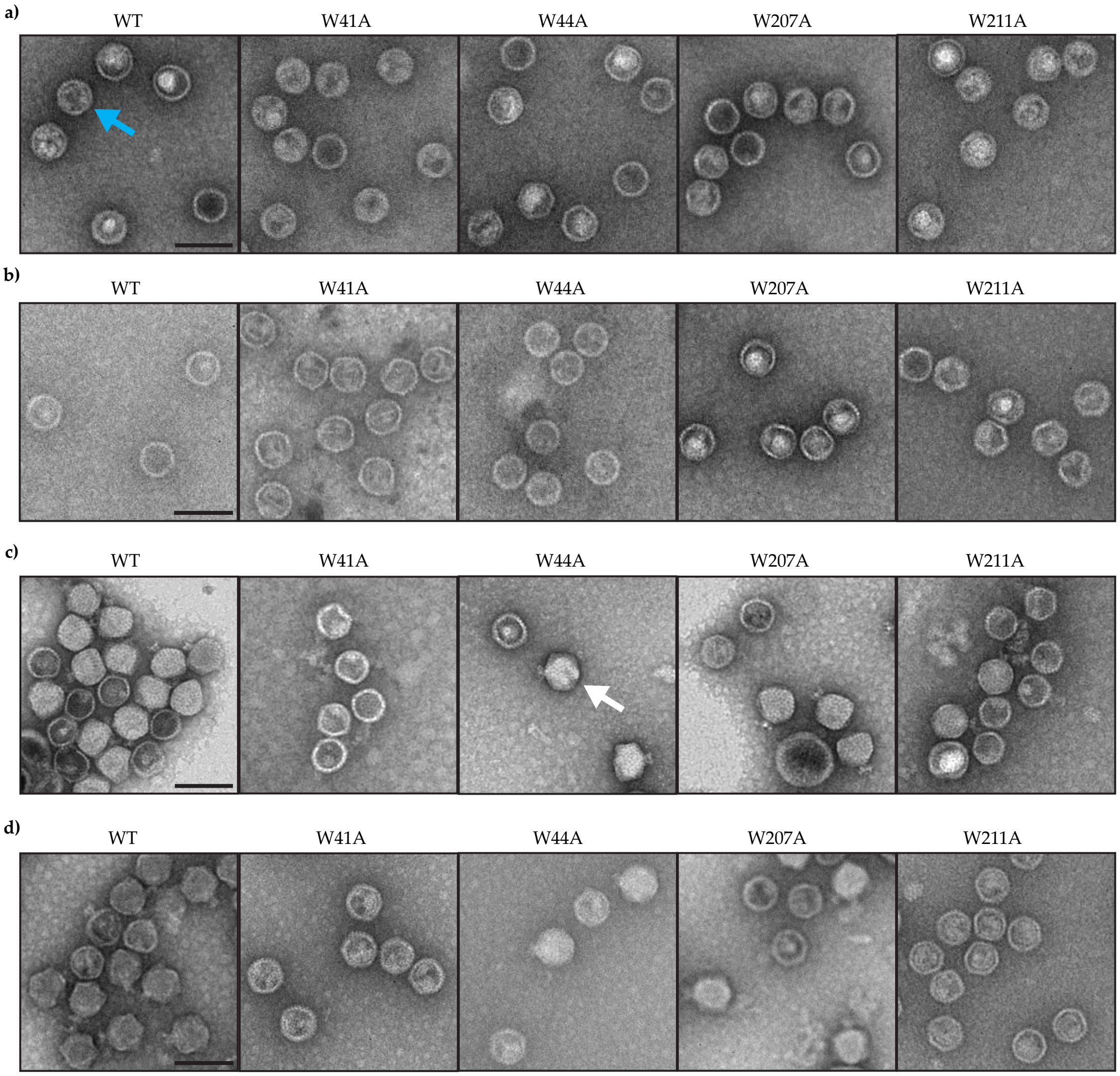

Figure S1. *In vivo* generated PCs and phages containing alanine-substituted portal proteins have normal morphologies. **(a)** and **(b)** TEM micrographs of the peak-PC containing fractions from 5-20% sucrose gradients of 30°C and 37°C cell lysates, respectively; **(c)** and **(d)** TEM micrographs of the phage fraction from 5-20% sucrose gradients of 30°C and 37°C cell lysates, respectively. The scale bar is 100 nm. The blue arrow shows a PC and the white arrow shows a phage.

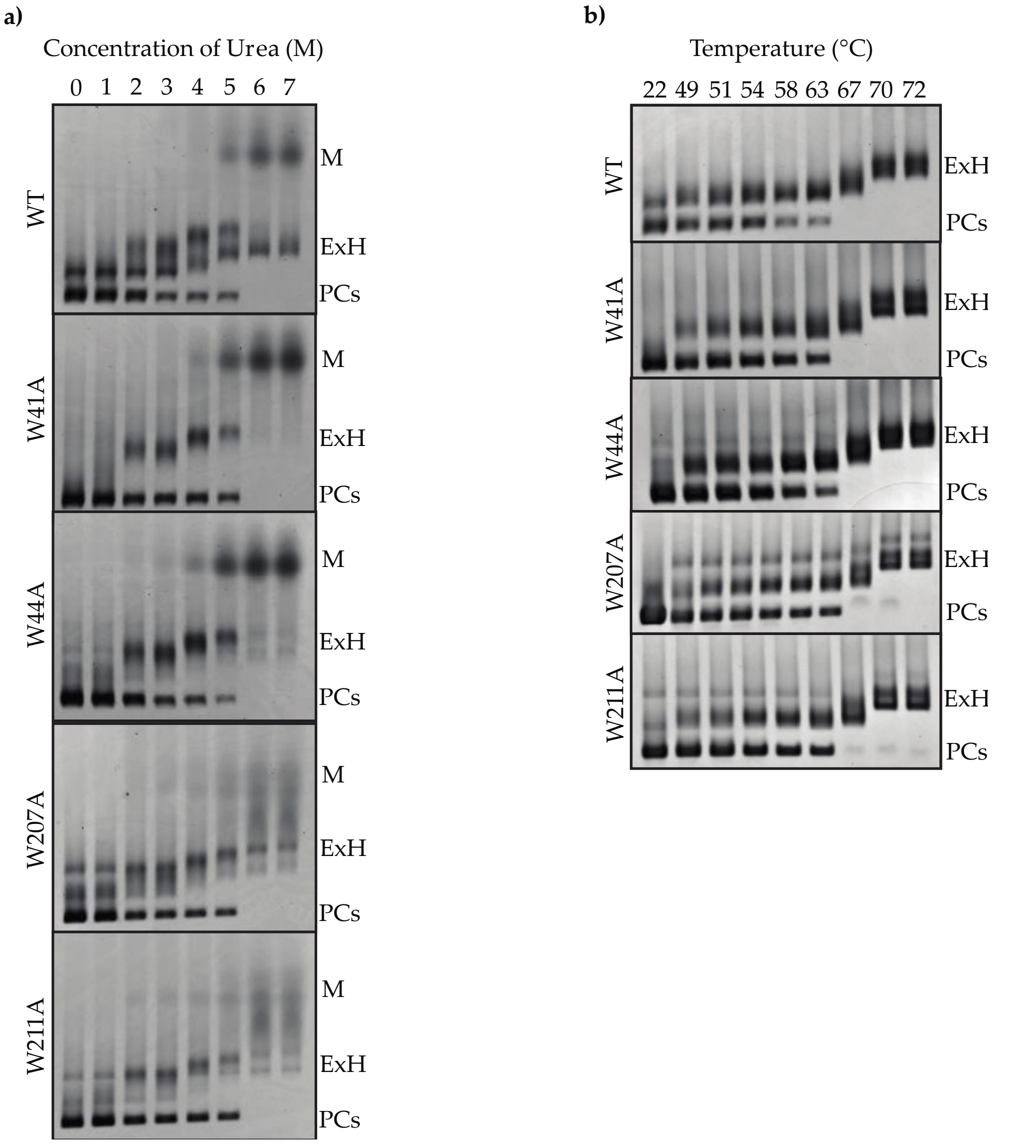

Figure S2. The alanine substitutions did not affect capsid stability or capsid expansion. **(a)** PCs assembled with WT, W41A, W44A, W207A and W211A portal proteins were incubated with varied concentrations of urea (0 - 7 M) to assess stability. Samples were run on a 1% native agarose gel. M, monomers. ExH, expanded heads. PCs, procapsids; **(b)** *In vitro* heat expansion of PCs assembled with WT and variant portal proteins. PCs were incubated at temperatures between 22°C – 72°C and run on a 1% native agarose gel.

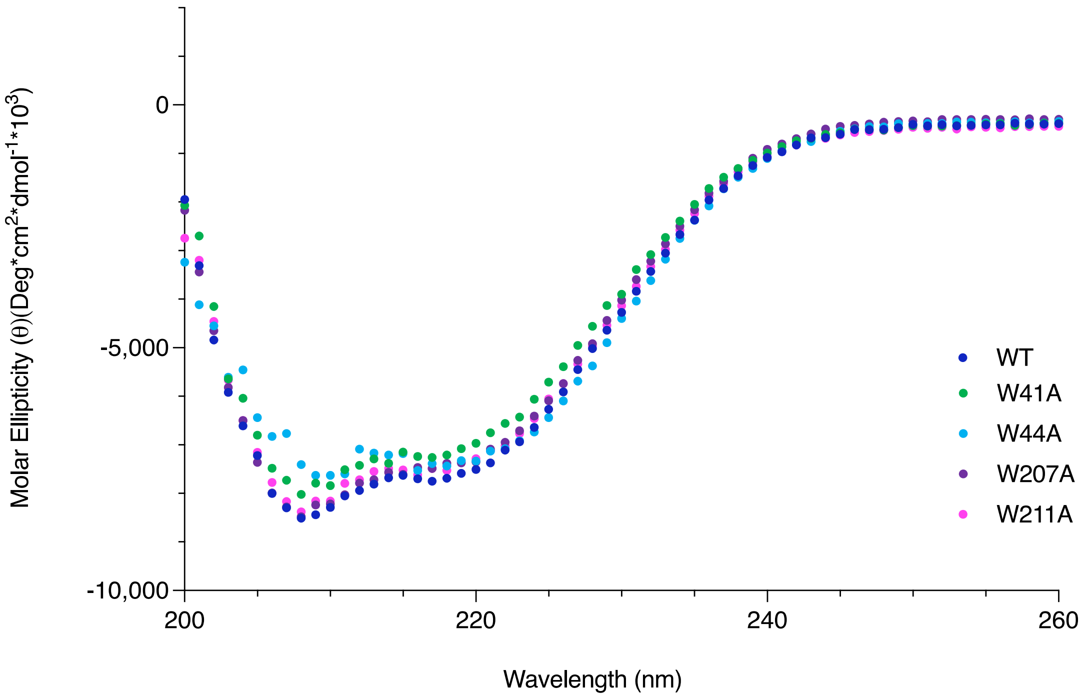

Figure S3. Circular dichroism of WT and alanine-substituted portal protein monomers. There is no significant difference between the WT portal monomers and variant portal monomers.
